# Organoids Increase the Predictive Value of *in vitro* Cancer Chemoprevention Studies for *in vivo* Outcome

**DOI:** 10.3389/fonc.2019.00077

**Published:** 2019-02-20

**Authors:** Rose N. Njoroge, Rajita J. Vatapalli, Sarki A. Abdulkadir

**Affiliations:** ^1^Department of Urology, Northwestern University, Feinberg School of Medicine, Chicago, IL, United States; ^2^Robert H. Lurie Comprehensive Cancer Center, Northwestern University Feinberg School of Medicine, Chicago, IL, United States; ^3^Department of Pathology, Northwestern University Feinberg School of Medicine, Chicago, IL, United States

**Keywords:** organoids, vitamin E, selenium, select, prostate cancer, anoikis, metabolism, extracellular matrix (ECM)

## Abstract

Epidemiological and preclinical data suggest that antioxidants are protective against prostate cancer whose pathogenesis has been linked to oxidative stress. However, the selenium and vitamin E Cancer Prevention Trial (SELECT), found no efficacy for selenium in reducing prostate cancer incidence while vitamin E was associated with an increased risk of the disease. These results have called in to question the models used in preclinical chemoprevention efficacy studies and their ability to predict *in vivo* outcomes. Chemoprevention agents have traditionally been tested on two dimensional monolayer cultures of cell lines derived from advanced prostate cancers. But as SELECT demonstrates, results from advanced disease models were not predictive of the outcome of a primary chemoprevention trial. Additionally, lack of cell-matrix interactions in two dimensional cultures results in loss of biochemical and mechanical cues relevant for native tissue architecture. We use recent findings in three dimensional organoid cultures that recapitulated the SELECT trial results to argue that the organoid model could increase the predictive value of *in vitro* studies for *in vivo* outcomes.

## Introduction

Prostate cancer (PCa) is the most commonly diagnosed non-cutaneous male malignancy in the United States. One in six men in the US is expected to be diagnosed with PCa in his lifetime. In 2018, an estimated 164,690 men will be diagnosed with PCa and 29,430 men will die from the disease (1). For men with local or regional disease at diagnosis, radical prostatectomy, or radiation therapy are effective treatments with a 100% 5-year survival rate (2). However, treatment related morbidity results in a poorer quality of life (3, 4). Moreover, 20–40% radical prostatectomy and 30–50% radiation therapy patients will experience biochemical recurrence within 10 years (5–7).

Early PCa growth and progression is driven by androgens making androgen deprivation therapy through surgical or medical castration, the standard treatment for advanced and metastatic PCa (8, 9). Eventually, however, there is progression to castration resistant disease, whose treatments confer a median overall survival benefit of less than 5 months (10–16). Moreover, widespread serum prostate-specific antigen screening has been associated with unnecessary biopsies, over diagnosis and overtreatment of indolent PCa (17). Given the drawbacks of screening, treatment associated morbidity and lack of effective treatments for advanced disease, preventing PCa is imperative.

The long natural history of PCa and its stepwise pathogenesis make it particularly amenable to prevention (2, 18, 19). Chemoprevention which uses natural, synthetic, or biological substances to reverse, slow or inhibit the initiation and progression of cancer, is an attractive public health strategy to reduce PCa incidence and treatment related morbidities (20). Androgen suppression using the 5α-reductase inhibitors, finasteride and dutasteride, in the Prostate Cancer Prevention Trial (PCPT) and REduction by DUtasteride of Prostate Cancer Events (REDUCE) randomized trials showed a 23 to 25% reduction in PCa relative risk (21, 22). These inhibitors were however not approved for PCa chemoprevention by the FDA as both were found to increase the risk of high-grade PCa with the possibility of increasing mortality (23). However, a long-term follow-up of the PCPT trial participants found a 43% relative risk reduction in low-grade PCa but no significant differences in overall survival or rates of high-grade disease (24). In spite of these findings, the two inhibitors are still not approved for PCa prevention (25).

## Oxidative Stress, a Promising Chemoprevention Target in PCa

Reactive oxygen species (ROS) are reactive molecules resulting from the partial reduction of molecular oxygen. Intracellular ROS comes from exogenous and endogenous sources (26). In small quantities, ROS take part in signal transduction by reversibly oxidizing protein thiol groups affecting numerous physiological processes (27, 28). High levels of ROS however indiscriminately damage cellular macromolecules (29). ROS can modify DNA bases, form DNA adducts, induce DNA cross-linking and cause DNA strand breaks (30). Oxidative DNA lesions that are not removed prior to DNA replication can lead to replication errors, mutations, and genome instability increasing the risk of carcinogenesis (31).

ROS also indirectly increase cancer risk if they cause lesions on tumor suppressor genes or inactivate negative regulators of oncogenes and DNA repair proteins (32, 33). Under physiological conditions, ROS accumulation is regulated by endogenous enzymatic and non-enzymatic antioxidant defense systems (26). Oxidative stress occurs when ROS levels outstrip these cellular antioxidant defenses (34). Oxidative stress has been linked to PCa development and progression. In Nkx3.1^−/−^ mutant mice, dysregulation in the expression of antioxidant and prooxidant enzymes led to oxidative stress and development of HGPIN while Nkx3.1/Pten double mutants which sustained more oxidative damage progressed to adenocarcinoma (35).

Products of oxidative damage; 8-hydroxy-2'-deoxyguanosine, 4-hydroxynonenal-protein-adducts, and nitrotyrosine were also detected in early prostatic tumorigenesis in the transgenic adenocarcinoma of the mouse prostate model (36). In the noble rat hormonal PCa model, administration of testosterone and β-estradiol triggered the expression of prooxidant enzymes and oxidative damage which induced stromal inflammation and dysplasia in the lateral prostate (37). For humans, several PCa risk factors including age, diet, inflammation and androgens are also associated with oxidative stress (38).

Additionally, key genetic and epigenetic changes in PCa have been shown to decrease the expression of genes relevant to prostatic redox homeostasis like GSTP1, Nrf2, NKX3.1, and NADPH oxidases (35, 39–41). Besides, transcription factors important for PCa like NK-κB, AP-1, HIF-1, and p53 are redox sensitive (42). Relative to benign prostate cells, human PCa cell lines display higher oxidative stress which is associated with a more aggressive phenotype (39). Moreover, as PCa progresses, patients display increasing levels of oxidative biomarkers including thiobarbituric acid reactive substances, 8-hydroxy-2'-deoxyguanosine and 4-hydroxynonenal-modified proteins concomitant with loss of antioxidant defenses (43–48).

## The Selenium and Vitamin E Cancer Prevention Trial (SELECT)

The links between oxidative stress and PCa pathogenesis are the basis of the notion that use of antioxidants can reduce risk of the disease. The SELECT trial tested the efficacy of the antioxidants selenium and vitamin E in reducing PCa incidence in 35,533 men over the age of 50 with an intended follow up of 12 years (49, 50). As part of the inclusion criteria, the men had to be free of a prior PCa diagnosis, have a non-suspicious digital rectal examination and a serum prostate specific antigen level of ≤ 4 ng/mL (50). SELECT, a phase III clinical trial, randomized participants to a daily dose of 200 μg L-selenomethionine and/or 400 IU α-tocopheryl acetate or placebo (49, 50). After 7 years of follow up, it was found that neither antioxidant reduced PCa incidence and that vitamin E was associated with a 17% increased risk of PCa compared to placebo (51).

## *In vitro* Models of Advanced Disease did not Predict Outcome of a Primary Chemoprevention Clinical Trial

Numerous *in vitro* studies suggest that vitamin E and selenium are antitumorigenic by inhibiting proliferation, altering redox homeostasis, inducing apoptosis, suppressing androgen receptor signaling, blocking inflammatory signaling among other mechanisms (52–92). SELECT's lack of efficacy and harmful effects however indicate that traditional *in vitro* models had a low predictive value for the clinical trial outcome (93, 94). In addition, the majority of *in vitro* chemoprevention studies predominantly use cell lines derived from advanced cancers including LNCaP, DU145, and PC3. These do not model disease initiation and they did not predict the outcome of a primary chemoprevention clinical trial like SELECT (94).

Cells at different stages of cancer progression likely respond differently to antioxidants due to differential regulation of prooxidant and antioxidant networks (95). ROS accumulation due to decreased antioxidant activity in premalignant lesions and early stage cancers could promote tumorigenesis through DNA damage and inactivating tumor suppressors (95). Advanced cancers however counter high ROS accumulation by increasing antioxidant activity through several mechanisms like upregulating NRF2 or increasing metabolic generation of reducing equivalents like NADPH mitigating oxidant damage (95).

## Increasing the Predictive Value of Preclinical Research by Using Three Dimensional Cell Cultures

*In vitro* testing of chemoprevention agents has historically utilized conventional two dimensional (2D) culture which does not recapitulate *in vivo* prostate biology. The human prostate is a multicellular secretory epithelium with a central lumen (96). The prostate's luminal layer is made of polarized columnar luminal epithelial cells which face the lumen and produce prostatic secretions (96). The prostate epithelium sits upon a basement membrane which is surrounded by a stromal compartment (97). Basement membranes are cell adherent sheet-like structured extracellular matrices (ECMs) that include laminins, collagens, proteoglycans, and glycoproteins (98). The ECM provides physical support and serves as a scaffold for tissue organization (99).

In addition, ECM attachment provides biochemical and mechanical cues necessary for anchorage dependent cell growth, proliferation, migration, and differentiation (99). ECM proteins bind growth factors, cytokines and chemokines creating spatial and temporal concentration gradients necessary for spatiotemporal coordination of cellular activity during tissue morphogenesis, wound healing, and chemotaxis (100). Integrins which link the ECM and the actin cytoskeleton also transmit external mechanical and internal actomysin contractile forces from and to the ECM altering cell migration, proliferation, and differentiation (101).

During normal epithelial glandular development, ECM–cell interactions provide contextual cues needed to maintain apico-basal polarity necessary for correct tissue architecture and function (102). The ECM aligns the mitotic spindle perpendicular to the apical–basal axis enabling symmetrical cell division in the plane of the monolayer which maintains tissue structure (102). ECM–cell interactions balance proliferation and cell death for tissue homeostasis and lumen morphogenesis; their disruption contribute to neoplastic transformation (99, 103). In contrast, cells in 2D culture lack physiological cell-cell and cell-matrix interactions leading to loss of native tissue architecture and function (104).

Additionally, attachment of the 2D monolayer to artificial surfaces affects cell morphology and signaling (105, 106). This has been attributed to the high failure rate of drugs screened using 2D *in vitro* cultures in clinical trials (107). Moreover, 2D cultures have a non-physiologically uniform distribution of oxygen and nutrients (108). Embedding cells in three dimensional (3D) matrices yields more physiological cell-cell and cell-matrix interactions. Organoids are 3D cellular aggregates derived from primary tissues/cells, induced pluripotent stem cells, or embryonic stem cells, which self-renew and self-organize to exhibit similar architecture and functionality as their tissue of origin (109). Organoids can also be derived from established cell lines (110).

Organoid self-renewing property lends itself well to a host of basic and translational research applications. It allows the expansion of cells from minuscule patient samples like solid tumor biopsies and circulating tumor cells for liquid biopsies, facilitating genetic profiling, drug screening, and potentially guiding personalized therapy (111, 112). Organoids can also be harnessed to study tissue development including embryonic development, lineage specification, tissue morphogenesis, and homeostasis and how these processes change during disease (113, 114). For example, organoids have been utilized to investigate the PCa cell of origin which could have prognostic and treatment benefits (115–118). Organoid co-cultures with stromal components will allow research in to the contribution of the tumor microenvironment to malignancy (119).

Prostate organoids phenocopy *in vivo* glandular morphology; they undergo polarization, lumen formation and produce prostatic secretions (115). Unlike 2D cultures, growth factor, nutrients and oxygen gradients in organoids yield heterogeneous cell populations like *in vivo* (107). Additionally avascular organoids better model solid tumors which are often poorly vascularized *in vivo*. Similar to tumors, cells at the center of large organoids are under various stresses including loss of ECM attachment and limits in the diffusion of nutrients and oxygen (120). This *in vivo*-like physiology of organoid cultures could increase the predictive value of preclinical research (107). Moreover, organoid cultures bridge the gap between 2D and animal models, that are challenging to generate for example the genetically engineered mouse models and which are affected by interspecies differences (121).

## Premalignant Organoids Recapitulate Outcome of the Select Trial

Effects of the mean concentrations of the SELECT agents attained in the blood plasma of the SELECT participants was evaluated in prostate organoids derived from normal, premalignant, and malignant prostate epithelial cells (122). The benign organoids were obtained from histologically and genetically normal prostate epithelium isolated from radical prostatectomy tissue (122). The premalignant organoids were derived from the untransformed RWPE-1 cell line which is immortalized with the E7 oncoprotein, modulating the activity of the retinoblastoma tumor suppressor (123, 124). The malignant organoids were cultured from the androgen responsive LNCaP cell line established from a lymph node PCa metastasis (125).

The vehicle-treated malignant organoids were devoid of lumens (filled morphology) a phenotype consistent with the acquisition of anchorage independent survival and loss of glandular differentiation (122). In contrast, normal epithelial cells require ECM attachment for proliferation and survival (99). This is partly because integrin ligation to the ECM regulates growth factor signaling and cell cycle progression (99). In these cells, ECM detachment activates a form of apoptotic cell death termed “*anoikis,”* which is Greek for homelessness (126). Anoikis clears cells in inappropriate locations preventing dysplastic growth (126). Anoikis also contributes to tissue homeostasis by eliminating cells without ECM contact in the lumen, hollowing glands (127).

Focal loss of ECM and integrin attachment in prostatic intraepithelial neoplasia lesions leads to cells proliferation within the lumen leading to defective glandular structures (128). In addition, PCa progression is marked by a gradual loss of glandular lumina (129). In cancer cells, oncogene activation overrides the requirement for ECM-adhesion leading to anchorage independent growth and resistance to *anoikis* (126). The SELECT agents decreased proliferation and increased cell death in the ECM distal cells at the center of the malignant organoids (122). This is consistent with reports that show antioxidant efficacy in PCa cell lines (60, 61). In contrast, the agents had no effect on the proliferation of benign organoids (122). Morphologically, the benign organoids had proper glandular structure with well-formed lumens which was not affected by vitamin E and/or selenium treatment (122).

The null effect of antioxidants on the benign organoids is consistent with SELECT where a fraction more subjects on vitamin E developed PCa compared to placebo (51). We posit that these individuals might have harbored initiated cells that progressed to neoplastic transformation with chronic vitamin E exposure. In agreement with this hypothesis, vitamin E alone or in combination with selenium but not selenium alone significantly increased the proliferation of premalignant RWPE-1 cell organoids (122). Therefore only results from the premalignant organoids recapitulated the clinical trial data from SELECT.

Our group has previously demonstrated that the antioxidant NAC causes premalignant prostatic epithelial hyperplasia in mice with prostate specific deletion of the Nkx3.1 tumor suppressor but not in wild type mice (130). In addition, polymorphisms in NKX3.1 were found to modulate PCa risk in men on the interventional arms of the SELECT trial (131). This points to the importance of the underlying genetic background in modifying the response to antioxidant supplementation. However, SELECT's inclusion criteria; prostate-specific antigen levels and a non-suspicious digital rectal exam, could not rule out the existence of initiating molecular aberrations (132).

Additionally, while the vehicle treated premalignant RWPE-1 organoids had more differentiated acinus structures and predominantly hollow lumens those treated with vitamin E had predominantly filled lumens (122). Microarray analysis of RNA extracted from vitamin E treated RWPE-1 organoids displayed significant downregulation of several integrins confirming the loss of matrix attachment (122). Vitamin E therefore increases cell survival in a low matrix environment (122).

## ECM Detached RWPE-1 Cells Have Deficient Glucose Metabolism Which Vitamin E Alleviates by Activating Fatty Acid Oxidation

Despite increased cell growth and survival, the vitamin E treated premalignant organoids had decreased expression of glucose transporters and several glycolytic enzymes implying glucose metabolism dysregulation (122). To further study metabolic changes in RWPE-1 cells under anchorage independent conditions *in vitro*, Poly-2-hydroxyethyl methacrylate (Poly-HEMA), suspension cultures were used (122). Poly-HEMA, a non-ionic polymer prevents ECM deposition on tissue culture plates and cell adhesion (126, 133). Additionally, growth of human epithelial cancer cells on poly-HEMA coated plates correlates with *in vitro* growth in soft agar the gold standard for measuring anchorage independent growth and tumorigenicity (134, 135).

Poly-HEMA cultures therefore provide a model for studying the regulation of anchorage-independent cell survival and growth for studies that are difficult to perform in organoids (136). Glucose is catabolized to Acetyl-CoA whose oxidative phosphorylation in the mitochondria produces reducing equivalents which mediate electron transfer in the electron transport chain generating the proton motive force that drives ATP synthesis (137). RWPE-1 poly-HEMA cultures had reduced glucose uptake and ATP levels confirming that loss of attachment jeopardizes cellular metabolism (122). Similar observations have been reported in detached MCF-10A benign breast epithelial cells (138).

**Figure 1:**
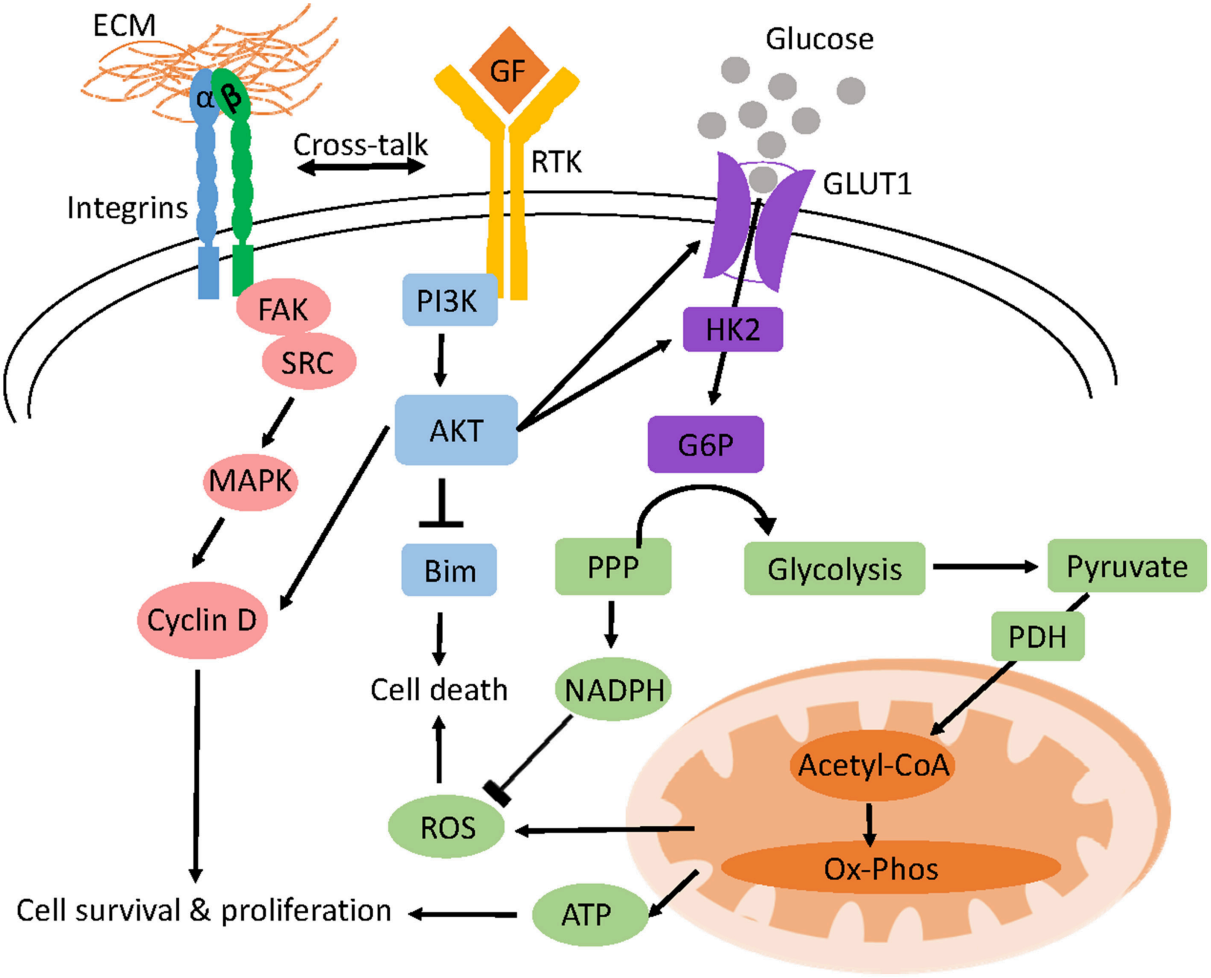
Model of anchorage-dependent regulation of cell survival and glucose metabolism. Adhesion of cells to the ECM activates integrins and receptor tyrosine kinase (RTK) signaling which triggers different pro-survival and proliferation pathways like AKT and MAPK. AKT inhibits BIM, a pro-apoptotic protein preventing *anoikis* and increases glucose uptake and glycolysis by upregulating the transcription of glucose transporters (GLUT1) and hexokinase 2 (HK2) respectively. Oxidative phosphorylation (OXPHOS) of pyruvate derived from glucose yields ATP for cellular function. Glucose shunted through the PPP pathway generates NADPH which prevents ROS induced cell death.

Altered metabolism after cell detachment has been attributed to the loss of integrin activation of the PI3K/AKT pathway which is a crucial regulator of glucose and glutamine uptake and metabolism (138). ECM-integrin contact leads to the recruitment of adopter proteins like talin and paxillin as well as signaling molecules like focal adhesion kinase (Fak) and small GTPases to form large macromolecular structures termed focal adhesions connecting the ECM and the actin cytoskeleton (139). Autophosphorylation of FAK (Y397) downstream of integrin signaling activates its kinase function leading to the activation of the SRC/MAPK and PI3K/AKT pathways which are crucial for progression through the G1/S checkpoint, cell survival and proliferation (Figure 1) (140, 141).

**Figure 2:**
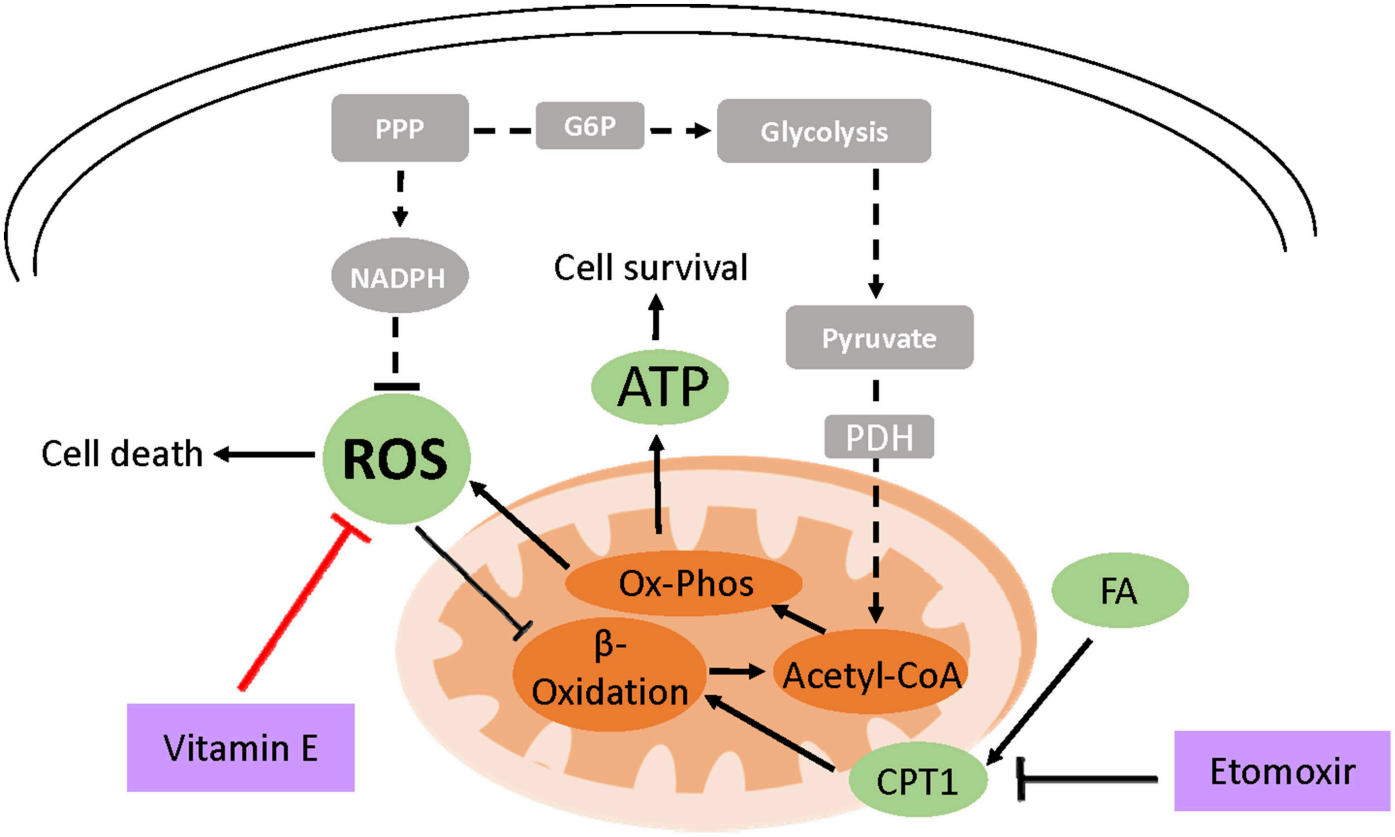
Model of vitamin E-mediated cell survival in ECM detached cells. Due to loss of integrin and PI3K signaling, cell detachment from the ECM results in the reduced expression of glucose transporters and low glucose uptake (shown in broken lines and gray boxes). This decreases ATP production and cell survival. Moreover, low NADPH generation through attenuated PPP flux leads to ROS accumulation which can induce cell death. ROS also inhibits fatty acid beta-oxidation (FAO) an alternative ATP generation pathway under glucose limiting conditions. However, treating detached cells with exogenous antioxidants like vitamin E neutralizes ROS which activates FAO increasing cell survival. Attenuating FAO using etomoxir which inhibits carnitine palmitoyltransferase (CPT1), FAO's rate limiting enzyme, abrogates vitamin E's ATP rescue ultimately diminishing cell survival.

Cross talk between integrins and receptor protein tyrosine kinases also activates the PI3K/AKT pathway downstream of the epidermal growth factor receptor (142). In contrast, the accumulation of multiple alterations allows cancer cells to circumvent such extracellular regulation enabling them to uptake nutrients constitutively (137). Glucose can also be shunted through the pentose phosphate pathway to generate the reducing equivalent NADPH and the nucleotide structural component, ribose-5-phosphate (137). In detached mammary cells low glucose uptake not only reduces ATP generation but also diminishes pentose phosphate pathway flux and NADPH production leading to ROS accumulation (Figure 2) (138).

Treatment of detached RWPE-1 cells with vitamin E rescued ATP generation but not glucose uptake (122). Similar findings have been reported in detached mammary cells treated with NAC or trolox, a soluble form of vitamin E (138). When glucose is scarce, cells can catabolize fatty acids for ATP generation. In Akt transformed glioblastoma, cells activate fatty acid oxidation to survive upon glucose withdrawal (143). It has been postulated that ROS inhibits fatty acid oxidation and hydrogen peroxide a form of ROS, has been shown to inhibit peroxisomal FAO (144–146). Pharmacological inhibition of fatty acid oxidation in vitamin E detached RWPE-1 cells abrogated the ATP rescue indicating that the antioxidant activates fatty acid catabolism (122).

Furthermore, fatty acid oxidation inhibition decreased cell survival and enhanced luminal clearance in vitamin E treated premalignant organoids indicating that antioxidants support anchorage independent cell survival (122). Moreover, other antioxidant-driven metabolic rescue mechanisms following loss of matrix attachment have also been described (147). In lung cancer spheroids, loss of matrix attachment upregulates glutamine reductive metabolism by cytosolic isocitrate dehydrogenase-1 to generate NADPH which is shuttled to inhibit mitochondrial ROS, enhancing cell growth (147). However, despite lowering ROS levels, selenium was not protumorigenic on the premalignant organoids (122). The effect of selenium on PCa risk has been shown to depend on baseline selenium status (148).

## Conclusions

The data showing antitumorigenic effects of antioxidants on malignant organoids replicates numerous two dimensional *in vitro* studies. However, 3D organoids starkly reveal differential effects of antioxidants along the prostate cancer evolution spectrum. In this system, vitamin E had a null effect in benign organoids and a pro-tumorigenic effect in premalignant organoids in a manner highly reminiscent of the SELECT trial results. These findings demonstrate that the use of preclinical models that better mimic *in vivo* conditions and disease stage yield data that is more relevant for clinical translation.

More broadly, organoid data in several cancers now show that neutralizing ROS promotes anchorage independent cell growth and survival implying that ROS accumulation can imperil detached cells (122, 138, 147). Anchorage independence facilitates cell growth and survival in non-native environments for example in metastasis (149). Therefore, identifying mechanisms that enable anchorage independence could offer clues on how to impede cancer metastasis (149). Finally, given the role that metabolism plays in carcinogenesis, preclinical studies ought to include metabolic endpoints when assessing potential chemopreventive agents.

## Author Contributions

SA and RN contributed to conception and design of the manuscript. RN researched supporting evidence and wrote the manuscript. RV and SA contributed to critical revision of content and figure design. SA secured grant funding.

### Conflict of Interest Statement

The authors declare that the research was conducted in the absence of any commercial or financial relationships that could be construed as a potential conflict of interest.
